# Characteristics of ciliary muscle profile in high myopes measured by swept-source anterior segment optical coherence tomography

**DOI:** 10.1371/journal.pone.0289135

**Published:** 2023-11-30

**Authors:** Hiu Yin Kwok, Hoi Yee Kwok, Tsz Nok Ng, Tsz-wing Leung, Byung Soo Kang, Chea-su Kee

**Affiliations:** 1 School of Optometry, The Hong Kong Polytechnic University, Kowloon, Hong Kong, SAR, China; 2 Research Centre for Sharp Vision, The Hong Kong Polytechnic University, Kowloon, Hong Kong, SAR, China; 3 Centre for Eye and Vision Research Limited, Hong Kong, SAR, China; Alexandria University Faculty of Medicine, EGYPT

## Abstract

**Objective:**

To characterize and compare the ciliary muscle thickness (CMT) between low and high myopes using swept-source anterior segment optical coherence tomography (AS-OCT).

**Methods:**

Forty visually healthy young Chinese adults aged 18–25 years were divided into two groups based on refractive errors: low myopia (n = 20, spherical-equivalent refractive error (SER) between −0.50 D to −3.00 D) and high myopia (n = 20, SER ≤ -6.00 D). Cycloplegic refractions were performed before axial length (AL) and CMT were measured using a partial coherence laser interferometer and an AS-OCT respectively. CMT was measured perpendicularly to the sclera-ciliary muscle interface at 1 mm (CMT_1), 2 mm (CMT_2), and 3 mm (CMT_3) posterior to the scleral spur, and at the location with maximal thickness (CMT_MAX).

**Results:**

High myopes demonstrated thicker CMT at 2 mm (CMT_2, p = 0.035) and 3 mm (CMT_3, p = 0.003) posterior to the scleral spur, but thinner maximal CMT (CMT_MAX, p = 0.005) than low myopes. The apical CMT_1 and CMT_MAX were also thinner in high myopes than in low myopes (both p< 0.001). CMT_MAX, apical CMT_1, and apical CMT_MAX correlated directly with SER and inversely with AL; in contrast, CMT_2 and CMT_3 showed inverse correlations with SER but direct correlations with AL.

**Conclusion:**

Our findings revealed significant differences in CMT between low and high myopes, with high myopes showing thicker CMT at 2 mm and 3 mm posterior to the scleral spur, but thinner maximal CMT. These results provide new evidence of the potential structural differences in ciliary muscles during myopia development and progression.

## Introduction

The ciliary muscle, a smooth muscle of the ciliary body, governs ocular accommodation by adjusting the shape and power of the crystalline lens through the contraction and relaxation of muscle tension [1]. A resilient ciliary muscle is crucial for maintaining clear vision across various viewing distances. Recently, there has been increased interest in ciliary muscle morphology due to its potential involvement in myopia (nearsightedness) development [2–4]. Myopia, a common, widespread, and progressive refractive error, is projected to affect half of the global population by 2050 [5]. Although no unified theory has fully elucidated the etiology of myopia, excessive near work has long been considered a primary risk factor [6–8]. In addition, myopic eyes typically exhibit a significant lag of accommodation [9–11], potentially resulting from inadequate ciliary muscle contraction control. The accommodation lag leads to hyperopic defocus, in which distant images are projected behind the retina. Such optical defocus could act as a visual error signal that accelerates myopic eye growth [12]. Additionally, the ciliary muscle’s anatomical proximity to the sclera suggests that biomechanical forces from ciliary muscle contractions may restructure the local arrangement of scleral collagen [13, 14], predisposing the eye to axial elongation and subsequent myopia development.

Morphology of the ciliary muscle is commonly quantified by its thickness, either as a whole [15] or at various locations posterior to the scleral spur [16–18]. The association between ciliary muscle thickness (CMT) and myopia remains an active area of research, as some studies have reported that increased myopia is correlated with thicker CMT, while others found no significant association (Table 1). These discrepancies may be attributable to differences in study design, CMT measurement methods, variations in subjects’ age range, and ethnicity [15–23]. The inconsistent findings underscore the necessity for further research on CMT in myopic eyes, particularly within specific population subgroups.

**Table 1:** Key findings of morphological changes of ciliary muscle in ametropia.

No.	Study	Methods	Total (N)	Age	SER (D)	High Myopes (N)	AL (mm)	Findings
**1**	Oliveira et al., 2005	UBM	75	NA (51.8±16.5)	-14.75 to 15.5 (-0.45±5.70)	14	15.5 to 30.1 (23.2±2.5)	1) CBT2 and CBT3 are thicker in myopes 2) SER and AL are correlated with CBT2 and CBT3
**2**	Bailey et al., 2008	AS-OCT	53	8 to 15 (11.8±2.31)	-6.00 to 3.44 (-1.13±2.26)	0	22.14 to 26.81 (23.81±1.15)	1) CBT2 and CBT3 are thicker in myopes 2) SER and AL are correlated with CBT2 and CBT3
**3**	Muftuoglu et al., 2009	UBM	19	11 to 44 (28.4±10.4)	-17.38 to -6.25 (-9.50±1.50)	19	25.16 to 30.21 (27.05±0.73)	1) CBT, CPT, and CMT are thicker in myopes 2) AL is correlated with CMT
**4**	Sheppard and Davies., 2010	AS-OCT	50	19 to 34 (25.8±4.5)	-9.50 to 0.88 (-2.00±2.62)	4	22.17 to 28.12 (24.49±1.13)	1) CMT2 is thicker in eyes with greater axial length (p = 0.06) 2) CML is higher in eyes with greater axial length
**5**	Jeon et al., 2012	UBM	31	19 to 35 (25.7±3.6)	-8.0 to 0.5 (-3.10±2.56)	5	23.75 to 27.15 (25.49±1.09)	1) CMT3 is thicker in myopes 2) CML is higher in myopes 3) SER and AL are correlated with CMT3 and CML
**6**	Pucker et al., 2013	AS-OCT	269	6 to 14 (8.7±1.5)	-4.01 to 7.76 (0.41±1.29)	0	19.96 to 25.97 (23.19±0.86)	1) CMT2 and CMT3 are thicker in myopes 2) CMT1 and CMTMAX are thicker in hyperopes 3) SER is correlated with CMT2, CMT3, and apical CMT1 & CMTMAX
**7**	Buckhurst et al., 2013	AS-OCT	62	18 to 40 (27.7±5.2)	-10.06 to 4.38 (-1.74±3.26)	NA	21.61 to 27.5 (NA)	1) CMT2 and CMT3 are thicker in myopes 2) SER and AL are correlated with CMT2 and CMT3
**8**	Kuchem et al., 2013	AS-OCT	62	21 to 40 (28.2±5.6)	-8.40 to 5.84 (-2.56±3.29)	8	21.36 to 26.51 (24.54±1.41)	1) SER is correlated with CMT1, CMT2, CMT3, CMTMAX, and apical CMT1 & CMTMAX
**9**	Kaphle et al., 2022	AS-OCT	70	18 to 27 (21.3±2.5)	-0.40 to -5.83 (NA)	0	21.96 to 26.96 (NA)	1) CMT1, CMT2, CMT3, CM50, CM75 are thicker in myopes 2) CML and CMLarc are higher in myopes 3) AL is correlated with CMT1, CMT3, CM25, CML, and CMLarc

SER = Spherical equivalent refractive error; AL = Axial length; CBT 2∼3 = Ciliary body thickness measured at 2∼3 mm posterior to scleral spur; CPT = ciliary process thickness; CMT1∼3 = Ciliary muscle thickness measured at 1∼3 mm posterior to scleral spur; CML = ciliary muscle length; CMLarc = ciliary muscle curved length; CMTMAX = maximum ciliary muscle thickness; Apical = thickness of the ciliary muscle’s apical portion; CM25∼75 = thicknesses at 25∼75% of the curved length of the ciliary muscle. Age, SER, and AL shown in range with mean and standard deviation.

Previously, anatomical changes in the ciliary muscle of ametropic eyes were measured using ultrasound biomicroscopy (UBM) [15, 17, 19]. This technology, however, requires direct contact with the ocular surface using a probe and yields relatively low-resolution images of the anterior ocular segment. Furthermore, anterior segment examinations using UBM often employ a fluid-filled scleral shell, necessitating patients to lie face-up, which may affect anterior segment geometry due to gravity [24, 25]. In contrast, anterior segment optical coherence tomography (AS-OCT), a non-invasive imaging technique with high-resolution capabilities for anterior eye structures, overcomes these limitations can better characterizes ciliary muscle morphology.

Despite the growing volume of research on ciliary muscles in myopic eyes, CMT in highly myopic eyes (–6.00 D or greater) remains underexplored. Previous studies, particularly those using AS-OCT, were limited to participants with low to moderate myopia [18, 20, 23] or included only a small number of highly myopic eyes (n < 8) [16, 17]. It is important to note that high myopia significantly increases the risk of multiple sight-threatening retinal diseases, such as glaucoma, cataract, myopic maculopathy, and retinal detachment [26]. In addition to its potential link to high myopia development, a comprehensive understanding of ciliary muscle anatomical changes holds significant clinical implications for presbyopia [27–29], glaucoma [30, 31], and intraocular lens (IOL) implantation [32–34]. Consequently, this study aims to characterize CMT in highly myopic eyes by comparing it with low myopes using a non-contact, high-resolution Fourier-domain swept-source AS-OCT [35].

## Methods

### Subjects

**Fig 1:**
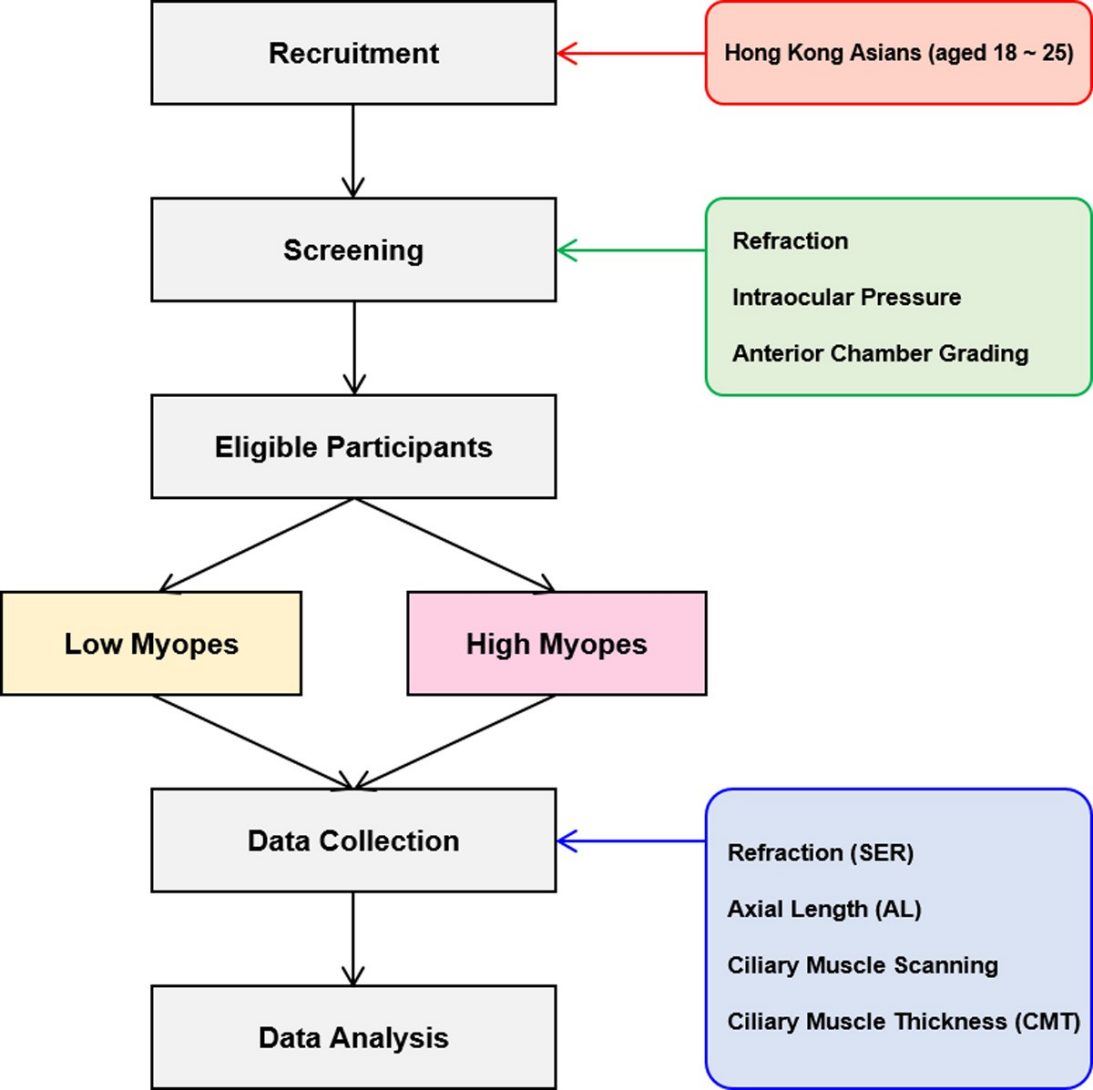
Flow chart depicting the experimental design.

Forty visually healthy young Chinese adults (aged 18 to 25 years) participated in this study between March 2021 and December 2021. The restricted age range aimed to avoid the potential effects of age in either developing or ageing eyes on CMTs [29, 36]. Participants were equally divided into two groups according to their refractive errors: 1) Low myopia group (n = 20): spherical-equivalent refractive error (SER) between –0.50 D to –3.00 D, astigmatism < 1.00 D; 2) High myopia group (n = 20): SER ≤ –6.00 D, astigmatism < 2.00 D. All participants had best-corrected distance visual acuity of 20/25 or better and anisometropia ≤ 1.00 D. Exclusion criteria included ocular or systemic diseases, strabismus, and a history of receiving ocular surgery or myopia interventions. A flowchart illustrating the study design is provided in Fig 1. The study adhered to the tenets of the Declaration of Helsinki, and the study design and protocols described below were reviewed and approved by the Human Subjects Ethics Sub-committee (HSEARS20210302005) of the Hong Kong Polytechnic University. Informed written consent was obtained from each participant before the study started.

### Procedures

Refractive errors and ocular biometric parameters were measured after cycloplegia. Participants underwent slit-lamp examination and non-contact tonometry (NT-530P, Nidek, Japan) measurements to determine suitability of cycloplegia administration. None of the participants had contraindications for cycloplegic agents, such as van Herick ratio < 0.25 or intraocular pressure > 21 mmHg. Gonioscopy was planned for individuals with a van Herick ratio < 0.5; however, all participants in the study had a van Herick ratio of 0.8 or larger. Two drops of 1% Tropicamide (Mydriacyl 1%, Alcon, USA), five minutes apart, were instilled to paralyze the ciliary muscle [37, 38]. This drug administration protocol has a comparable cycloplegic effect to 1% cyclopentolate [39–41]. Objective refraction was performed using an autorefractor (ARK-510A, Nidek, Japan) 30 minutes after administering the first eye drop of cycloplegia. The refractive error was then confirmed with subjective refraction using the maximum plus maximum acuity as the endpoint [42]. Spherical-equivalent refractive error (*SER*) was calculated according to the spherical (*S*) and cylindrical (*C*) errors:

$$SER=S+\frac{1}{2}C.$$

Axial length (AL) was measured from the anterior corneal apex to the retinal pigment epithelium layer using partial coherence laser interferometry (IOLMaster, Zeiss, Germany). Five consecutive readings were acquired and averaged for each participant.

#### Ciliary muscle thickness (CMT) measurement

**Fig 2:**
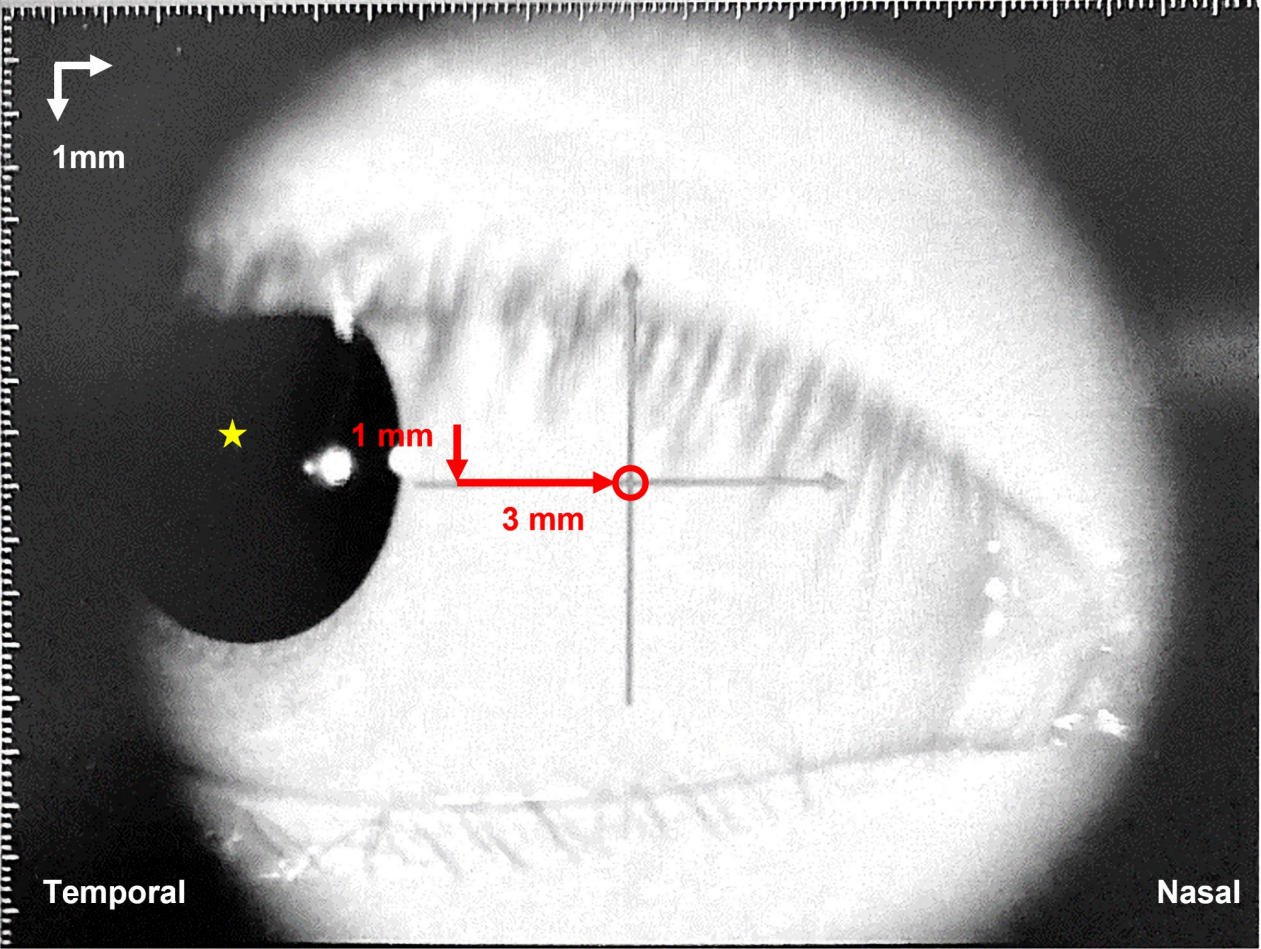
A representative image of nasal ciliary muscle captured by the AS-OCT. Ciliary muscle thicknesses at the nasal side were measured at the location (red circle) 1 mm below the pupil center (yellow star) and 3 mm nasally from the limbus.

A swept-source Anterior Segment Optical Coherence Tomographer (AS-OCT, Casia SS-1000, Tomey Corporation, Japan) was used to characterize the ciliary muscle profile following the completion of cycloplegic refraction (approximately 40 minutes after the first drop of cycloplegia instilment). Participants, who had undergone cycloplegia, were instructed to fixate on an internal target located at the temporal visual field inside the instrument to enable locating and scanning the anterior segment at the nasal side (about 1 mm below the pupil center and 3 mm nasally from the limbus; Fig 2, area circled in red). The nasal CMT was chosen for measurement because the scleral spur could be more easily identified than the temporal side [43]. Images were acquired under the 2D Angle HD mode, with a sampling resolution of 2048-line A-scan per image and a cross-sectional scan range of 8 mm x 8 mm. Three consecutive images were captured and analyzed.

The Casia SS-1000 AS-OCT has been previously validated and demonstrated to have excellent intra-device repeatability for measuring anterior chamber depth, angle-to-angle distance, pupil diameter, and crystalline lens rise [44]. However, the repeatability of CMT measurements has not been assessed. To confirm the repeatability of CMT measurements, an additional set of images were collected within the same day of the visit. After the first set of image acquisition, participants were asked to lean back from the device. The same examiner repeated the procedures described above to realign the eye position with AS-OCT and re-measure the CMT.

**Fig 3:**
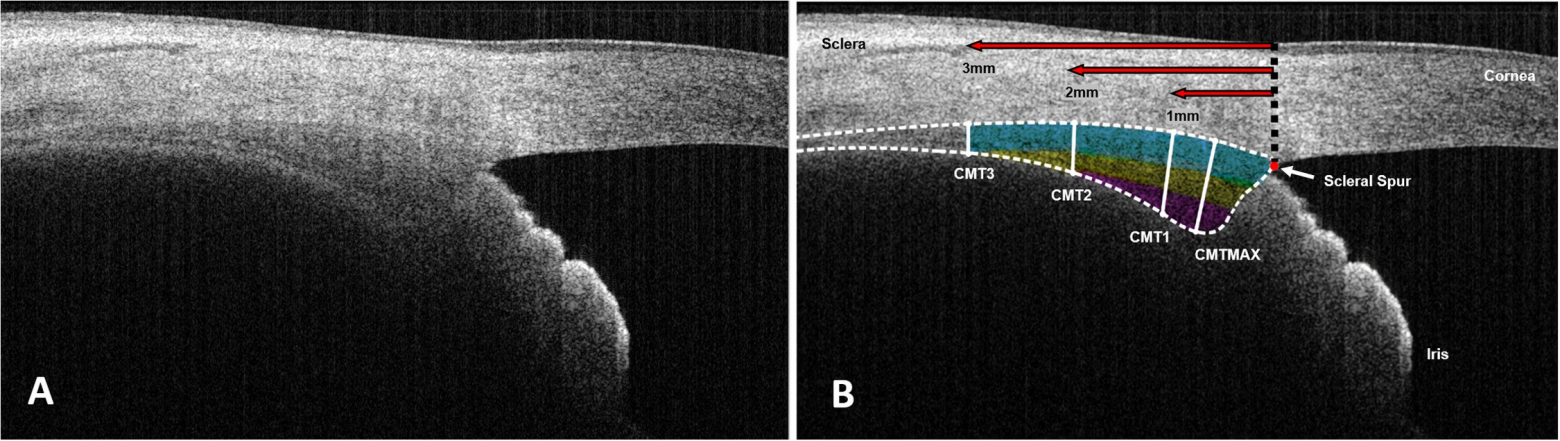
Representative images of the nasal ciliary muscle captured by AS-OCT. (A) The raw AS-OCT image. (B) The processed AS-OCT image. The dashed line encloses the region of interest, while the solid lines indicate the CMT measured at different locations: 1 mm (CMT_1), 2 mm (CMT_2) and 3 mm (CMT_3) posterior to the scleral spur or at the point of maximal thickness (CMT_MAX). Color-shaded areas indicate individual CMT regions: blue (CMT_3), yellow (CMT_2), and pink (CMT_1 and CMT_MAX).

The AS-OCT images obtained were subjected to processing using a protocol similar to that employed by previous studies [18, 21, 22]. The image analysts were masked to the refractive states of the subjects to mitigate the effect of subjective measurement bias. First, the raw AS-OCT images were processed using the “Sharpen” function in ImageJ (National Health Institute, USA) [45], enhancing contrast and accentuated details to facilitate clear differentiation of the ciliary muscle-sclera boundary (Fig 3A). Subsequently, the ImageJ scale was calibrated as follows: (a) distance in pixels = 100; (b) known distance = 1; (c) unit of length = mm; (d) decimal place = 5. This calibration ensured 1:1 correspondence between the AS-OCT image and physical measurements, thereby aiding in accurate determination of CMT at a later stage. Afterwards, a non-destructive grid of lines, covering an area of 1 mm², was created using an ImageJ plugin. The AS-OCT images were rotated to optimize alignment with the scleral contour. Manual measurements of CMT were then performed perpendicular to the grid at various locations, with each measurement repeated thrice to ensure accuracy. The position of the scleral spur was first defined in the AS-OCT images by tracking the boundary between the ciliary muscle fibers and the sclera until it reaches the anterior chamber (Fig 3B) [46]. The CMT was then measured perpendicularly to the sclera-ciliary muscle interface at 1 mm (CMT_1), 2 mm (CMT_2), and 3 mm (CMT_3) posterior to the scleral spur and at the location with the maximal thickness (CMT_MAX). The thickness of the ciliary muscle’s apical portion, which is mainly composed of circular fibers (and partially radial fibers), was calculated at 1 mm posterior to the scleral spur and at the location demonstrating maximal CMT:

ApicalCMT_1=CMT_1–CMT_2

ApicalCMT_MAX=CMT_MAX–CMT_2

The CMT and apical fiber thickness obtained from the three consecutive captured images were averaged for each measurement for further analyses.

## Statistical analysis

All statistical analyses were performed using IBM SPSS (Version 26, IBM, Armonk, NY, USA). To evaluate intra-session repeatability of AS-OCT in CMT measurement, paired *t*-test with Bland-Altman analysis (95% limits of agreement: bias ± (1.96 x SD of differences)) were applied to compare the differences between the two sets of images collected from the each participant [47]. The differences in CMT between the low and high myopic groups were compared by unpaired *t*-tests or Mann-Whitney *U-*tests after verifying data normality with the Shapiro-Wilk test. Spearman’s correlation coefficient analysis was performed to test the relationship between CMTs and ocular biometric parameters (SER and AL). The significance level for all tests was set at 5%.

## Results

### Demographic information

Table 2 summarizes the demographic information for the low and high myopic groups. As expected, high myopes had SER −6 D and axial length 2.7 mm longer than the low myopic group (unpaired *t*-test, all p< 0.001). However, there were no significant differences in age (Mann-Whitney *U-*test, p = 0.904) and gender proportion (Chi-squared χ^2^ test, p = 0.204) between the groups.

**Table 2:** Demographic characteristics of low and high myopes.

	Low myopes	High myopes	P-value
**Subject Number**	20	20	-
**Age (yrs)**	20.50 ± 2.07	20.60 ± 2.16	0.904
**Gender (male:female)**	13:7	9:11	0.204
**SER (D)**	−1.67 ± 0.73 (−0.625 to −3.00)	−7.60 ± 1.36 (−11.00 to −6.00)	0.001
**AL (mm)**	24.14 ± 0.49 (22.82 to 25.07)	26.83 ± 0.60 (25.57 to 28.47)	0.001

SER = Spherical-equivalent refractive error; AL = axial length. Data are presented in mean±SD with ranges indicated in brackets. Unpaired *t*-tests were used except for Age (Mann-Whitney *U*-test) and Gender (Chi-squared χ^2^ test).

### Repeatability of AS-OCT

**Fig 4:**
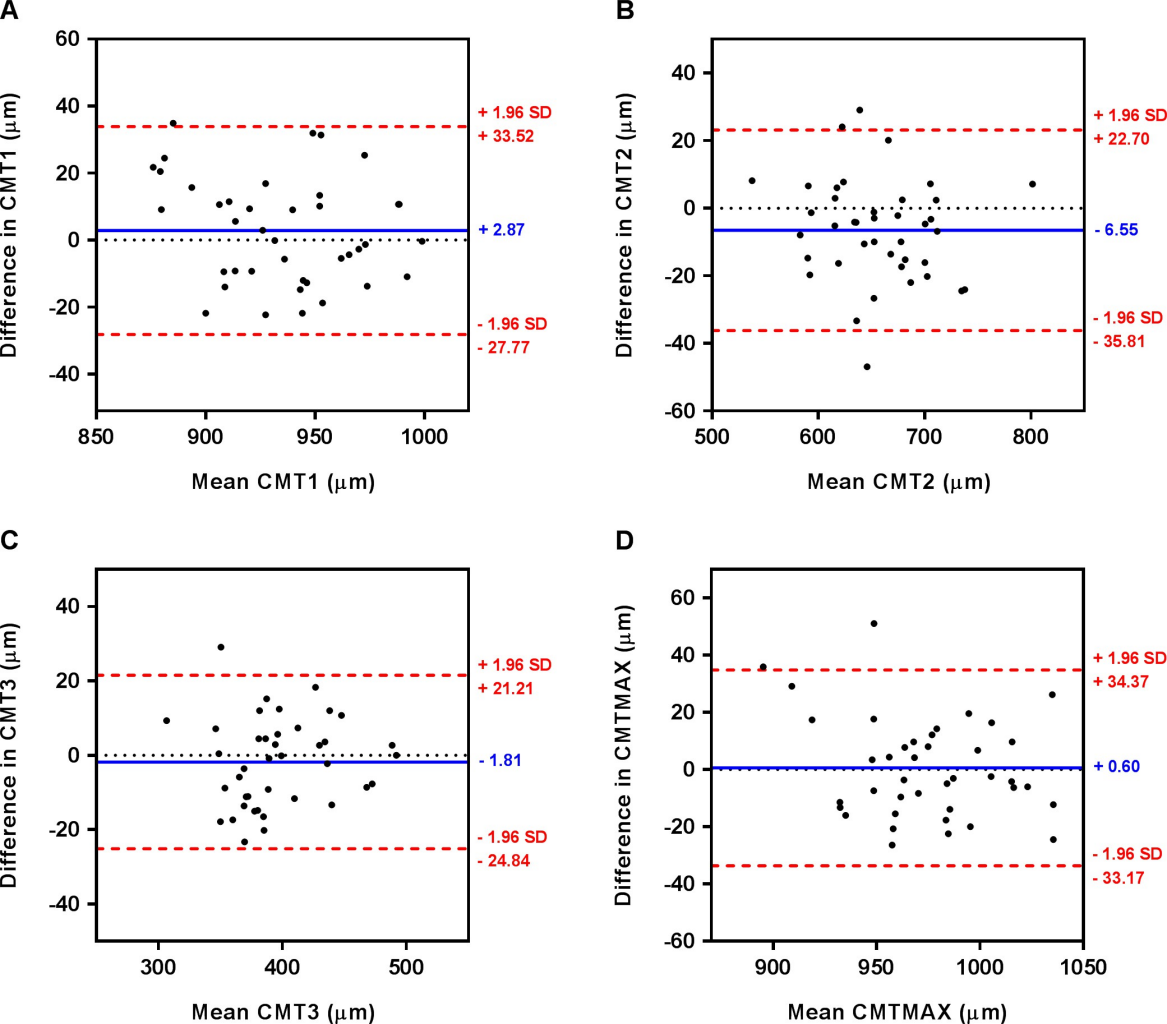
Bland-Altman plots showing the agreement between the two sets of regional CMTs. In each plot, the solid blue line and dotted red lines represent the mean difference and the 95% limits of agreement, respectively.

All AS-OCT image acquisitions were repeated twice in the same visit and the intra-session repeatability in CMT measurement was satisfactory (Table 3). There were no significant differences between the two sets of CMTs measurements (paired *t*-tests, all p≥ 0.26), except for CMT_2 (mean difference: −6.55 μm, paired *t*-test, p = 0.009) and apical CMT_1(mean difference: −9.42 μm, paired *t*-test, p = 0.014). The Bland-Altman analysis showed no systematic trend for all regional CMTs (Fig 4).

**Table 3:** Mean differences and 95% limits of agreements (LoA) between the two sets of CMT measurements.

Measurements (μm)	Mean Difference (95% CI)	95% LoA	P-value
**CMT_1**	2.87 (−2.19 to 7.94)	−27.78 to 33.52	0.258
**CMT_2**	−6.55 (−11.38 to -1.72)	−35.81 to 22.71	0.009
**CMT_3**	−1.81 (−5.62 to 2.00)	−24.84 to 21.21	0.341
**CMT_MAX**	0.60 (−4.98 to 6.18)	−33.17 to 34.38	0.829
**Apical CMT_1**	9.42 (2.02 to 16.82)	−35.34 to 54.19	0.014
**Apical CMT_MAX**	7.15 (−0.10 to 14.41)	−36.75 to 51.06	0.053

CMT = ciliary muscle thickness at 1 mm (CMT_1), 2 mm (CMT_2) and 3 mm (CMT_3) posterior to the scleral spur or at the point of the maximum thickness (CMT_MAX); Apical CMT_1 = CMT_1 –CMT_2; Apical CMT_MAX = CMT_MAX–CMT_2; CI = confidence interval; Paired *t*-tests were used to test the difference between the sets of repeated measurements.

### Ciliary muscle thickness in high myopes

As shown in Table 4, all regional CMT readings except CMT_1 (unpaired *t*-test, p = 0.108) showed significant differences between low and high myopes. The high myopic group had thicker CMT at 2 mm (CMT_2) and 3 mm (CMT_3) posterior to the scleral spur by 34.74±68.50 μm (p = 0.035) and 40.47±51.89 μm (p = 0.003), but thinner maximum CMT (CMT_MAX) by 31.82±56.10 μm (p = 0.005) than the low myopic group. The apical CMT_1 and CMT_MAX were also thinner in high myopes than in low myopes, by 53.24±44.72 μm and 66.55±51.80 μm (both p< 0.001), respectively.

**Table 4:** Comparisons of CMTs between low and high myopes at different locations.

Parameters	Low Myopes	High Myopes	High–Low Myopes	P-value
CMT_1	942.93±36.36 (925.92 to 959.95)	924.43±34.81 (908.14 to 940.72)	−18.51±55.44	0.108
CMT_2	642.56±41.66 (623.06 to 662.05)	677.3±57.4 (650.43 to 704.16)	34.74±68.50	0.035
CMT_3	377.09±31.27 (362.45 to 391.72)	417.56±40.48 (398.61 to 436.50)	40.47±51.89	0.003
CMT_MAX	989.9±33.13 (974.39 to 1005.40)	958.09±35.06 (941.67 to 974.49)	−31.82±56.10	0.005
Apical CMT_1	300.38±42.1 (229.54 to 264.73)	247.14±37.6 (280.67 to 320.08)	−53.24±44.72	<0.001
Apical CMT_MAX	347.34±38.88 (329.14 to 365.53)	280.79±42.82 (260.74 to 300.83)	−66.55±51.80	<0.001

CMT = ciliary muscle thickness at 1 mm (CMT_1), 2 mm (CMT_2) and 3 mm (CMT_3) posterior to the scleral spur or at the point of maximal thickness (CMT_MAX). Data are presented as mean±SD (μm) with 95% confidence intervals indicated in brackets. Unpaired *t*-tests were used except for the CMT_3 (Mann-Whitney *U*-test).

**Fig 5:**
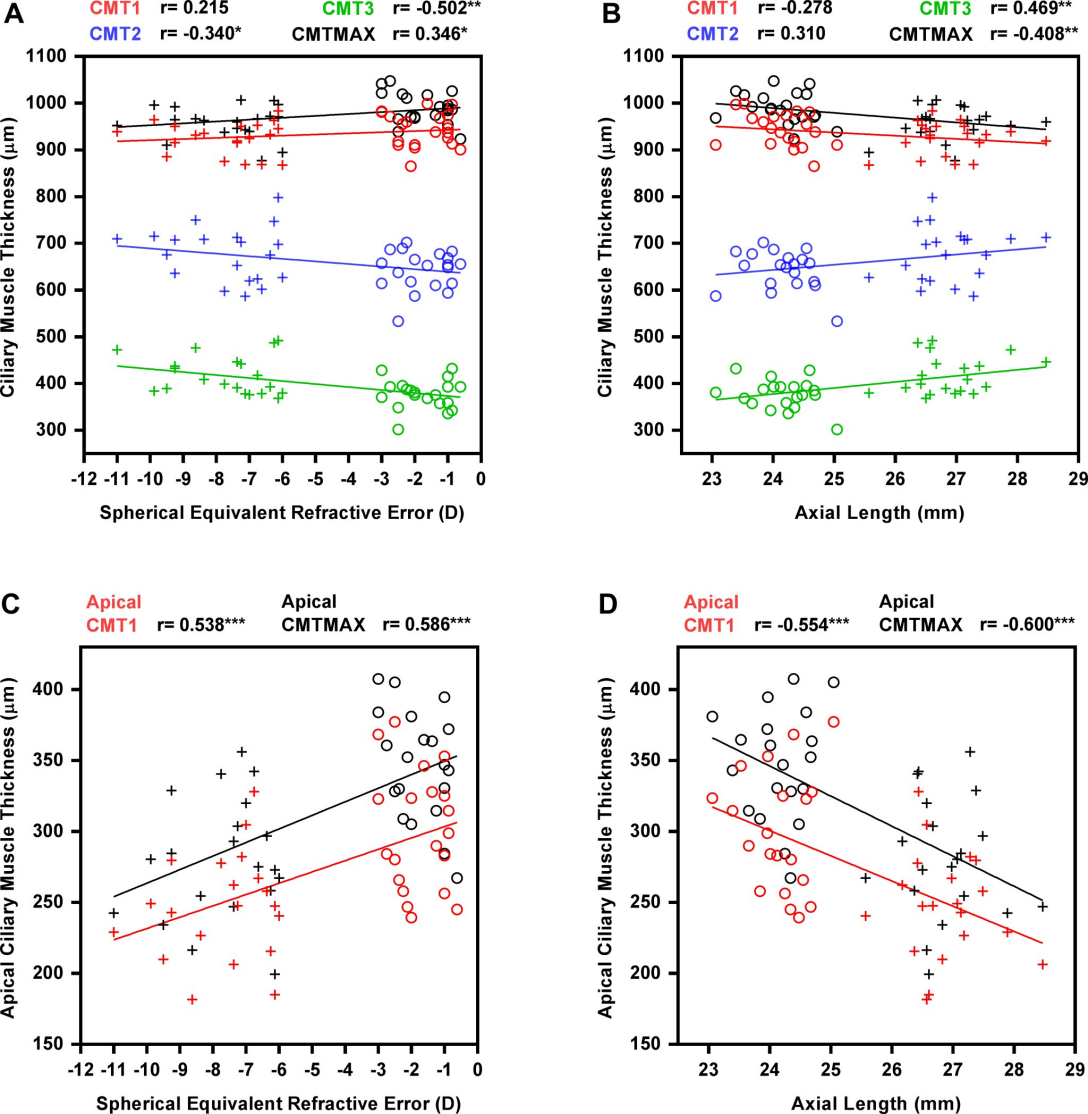
Correlations between CMTs and biometric parameters at different locations. CMTs (A&B) and apical CMTs (C&D) were plotted against SER (Left) and AL (Right). Circle and cross symbols represent low and high myopes, respectively. Red: CMT_1, blue: CMT_2, green: CMT_3, black: CMT_MAX. Pearson’s r is presented. *p<0.05, **p<0.01, ***p<0.001.

The regional CMTs, except CMT_1 (Pearson’s r< 0.215, p> 0.08), were significantly correlated with SER and axial length (Fig 5A and 5B). CMT_MAX, apical CMT_1, and apical CMT_MAX were correlated directly with SER (all Pearson’s r≥ 0.346, p< 0.05) and inversely with axial length (all Pearson’s r≥ −0.408, p< 0.01), indicating that eyes with higher myopia and longer axial length were associated with thinner maximal ciliary muscle thickness and apical fiber thickness. In contrast, CMT_2 and CMT_3 were inversely correlated with SER (Pearson’s r = −0.340 & −0.502, p = 0.03 & 0.001) and axial length (Pearson’s r = 0.310 & 0.469, p = 0.051 & 0.002).

## Discussion

This study characterized and compared regional ciliary muscle thicknesses between low and high myopes using a high-resolution AS-OCT. The findings revealed that compared to low myopes, the ciliary muscle in highly myopic eyes exhibited reduced thickness at the apical and maximum thickness regions (CMT_MAX, apical CMT_1, and apical CMT_MAX), and increased thickness posteriorly (CMT_2 and CMT_3) (see Table 4). An earlier study employing UBM also reported a generally thicker ciliary muscle in highly myopic eyes: however, due to the limited image resolution, the analysis focused on the ciliary muscle as a whole rather than conducting a comprehensive assessment of regional morphology [15]. Consistent with a subsequent study employing a higher resolution AS-OCT [16, 18, 20–23], our results identified a thicker CMT_2 and CMT_3 in high myopes than low myopes. The significant correlations observed between CMT_2 and CMT_3 with SER and axial length confirm that an increase in ciliary muscle thickness was associated with higher myopia and increased axial length, which are generally in line with other AS-OCT studies that primarily examined low and moderate myopes (Bailey et al. [20], Sheppard and Davies (only CMT2) [16], Jeon et al. (only CMT3) [17], Pucker et al. [18], Buckhurst et al. [21], and Kaphle et al. [23]).

While myopic eyes consistently exhibit a thickening of the posterior ciliary muscle, the variations in maximum ciliary muscle thickness and apical ciliary muscle thickness in association with myopia have revealed inconsistencies across studies. In concordance with the current findings, Pucker et al. [18] and Kuchem et al. [22] found that apical CMT_1 and CMT_MAX were thinner, rather than thicker, in eyes with higher myopia. Conversely, some studies found no significant correlation between apical ciliary muscle thickness and myopia or axial length. It is essential to recognize the differences in the study populations of these studies. Pucker et al. [18] focused exclusively on children (aged 6–14 years) with low to moderate myopia, while Kuchem et al. [22]. investigated anisometropic young adults, including only eight participants with high myopia in one eye. Our research contributes new evidence that shows the apical ciliary muscle was thinner while the posterior ciliary muscle was thickened in highly bilaterally myopic young adults, compared to age- and gender-matched low myopes.

Myopic eyes consistently display morphological changes in the ciliary muscle; however, the causal relationship with myopia development remains uncertain. It was initially anticipated that ocular expansions in myopic eyes would stretch the ciliary muscle, resulting in a more elongated and thinner configuration [48]. Notably, the current findings revealed that the thinning of the ciliary muscle occurred only at the apical or maximum thickness regions, which are less likely to be subjected to stretching. In contrast, the ciliary muscle at the posterior segment was thickened, as corroborated by other studies. The consistency of these observations suggests that mechanical stretching during myopia development may not directly influence ciliary muscle morphology, particularly in the posterior segment. Alternatively, the morphological changes in the ciliary muscle of high myopes may be attributable to relocation of the ciliary muscle to a more posterior position due to axial elongation [16]. This process could result in a relatively thinner apical and thicker posterior ciliary muscle.

It has also been hypothesized that an eye with thicker posterior ciliary muscles may be more susceptible to myopia development. The thickened ciliary muscle could generate higher mechanical resistance, constraining equatorial expansion during emmetropization. The disproportionate axial elongation [49] may account for the relatively prolate posterior retinal contour frequently observed in myopic eyes [50]. Such a prolate retinal contour leads to relative hyperopic defocus in the peripheral visual field [49, 50]. The relatively peripheral hyperopic defocus, which is recognized as a visual error signal for axial myopic eye growth, may increase the risk for myopia development [12, 51–53] (note, however, contradictory findings in Mutti et al. [49]). Furthermore, ciliary muscle hypertrophy may somewhat impair contractile function, leading to a more significant lag of accommodation. As a result, the hyperopic defocus during near work due to accommodative lag may also instigate and accelerate myopia development [20]. However, other studies have reported contradictory findings [9, 35, 54, 55].

To the best of our knowledge, this is the first study to have examined the regional anatomical variations in ciliary muscles of highly myopic patients by employing state-of-the-art imaging modalities. Previously applied techniques, such as UBM, exhibit commendable repeatability [15, 56]; however, the contemporary swept-source AS-OCT has garnered interest due to its capacity to generate high-resolution cross-sectional anterior segment images without touching the cornea [57]. The earlier AS-OCT model (time-domain Visante AS-OCT, Zeiss, Germany) significantly contributed to understanding the correlation between ciliary muscle thickness and myopia [21], but suffered from drawbacks, including a sluggish image acquisition rate [58]. In contrast, the Casia SS-1000 AS-OCT, a first-generation swept-source Fourier-domain OCT instrument, facilitated rapid anterior segment imaging in a single session [35]. The findings of the current study indicate that the Casia SS-1000 AS-OCT could measure ciliary muscle thickness with high repeatability (Table 3 and Fig 4). Despite a significant difference of 6.55 μm between the two sets of repeated measurement for CMT_2, this discrepancy seemingly exerts no influence on identifying substantial differences in ciliary muscle thicknesses between high and low myopes, which were > 18.51 μm (Table 4).

In conclusion, our results support the notion that ciliary muscle thicknesses display variations between low and high myopes. However, certain limitations in the current study warrant consideration for future experiments. First, although the repeatability of AS-OCT is adequate for discerning CMT differences between low and high myopes, it should be noted that the range of 95% LoA was considerable in some instances (e.g., CMT_2: −35.81 μm to 22.71 μm, Table 3). This may affect the reliability of CMT comparisons for small between-group differences (i.e., the difference in CMT between low and high myopes: 18.51 μm to 40.47 μm, see Table 4). Second, the resolution of Casia AS-OCT may introduce variability in CMT measurements. It was observed that image resolution varied across ciliary muscle regions, with the scleral spur and the ciliary muscle apex appearing more distinct in the posterior regions compared to the anterior regions. Thus, determining the region for CMT_1 and CMT_MAX measurements posed challenges. Third, this study did not assess inter-examiner repeatability. The accuracy of manual pixel coordinate selection for the scleral spur and the ciliary muscle margin could potentially influence the repeatability of CMT readings. Further evaluation of inter-session, inter-examiner, and intra-examiner repeatability is necessary. Fourth, the methodology employed for segmenting regions for CMT measurements does not account for the possibility of varying ciliary muscle length depending on refractive errors [16]. Incorporating proportional CMT measures may be required to ensure comparisons are made in similar regions of ciliary muscles. Fifth, a recent study observed alternations in CMT after administering 1% atropine [59]. However, considering the varying accommodative demands between the low and high myopic groups when fixating on the fixation target during measurements, the current study employed two drops of 1% tropicamide to minimize the potential accommodation fluctuations and mitigate their impact on the comparison of CMT between groups [2, 3]. Although the current study utilized a different cycloplegic agent, the ciliary muscle morphology presented may still not fully represent its natural state. It underscores the importance of a comprehensive evaluation of the potential influence of various cycloplegic agents on ciliary muscle morphology. Finally, this study offers only cross-sectional data on the ciliary muscle morphology in high myopes with a limited sample size derived from a Chinese population. Thus, cautions should be paid when extrapolating these findings to other highly myopic populations. Longitudinal studies are also warranted to investigate the mechanism and role of ciliary muscles in myopia development.

## Supporting information

S1 Data (XLSX)
